# Hedgehog Signaling Regulates Telomerase Reverse Transcriptase in Human Cancer Cells

**DOI:** 10.1371/journal.pone.0075253

**Published:** 2013-09-25

**Authors:** Tapati Mazumdar, Ranjodh Sandhu, Maha Qadan, Jennifer DeVecchio, Victoria Magloire, Akwasi Agyeman, Bibo Li, Janet A. Houghton

**Affiliations:** 1 Department of Cancer Biology, Lerner Research Institute, Cleveland Clinic, Cleveland, Ohio, United States of America; 2 Department of Biological, Geological and Environmental Sciences, Center for Gene Regulation in Health and Disease, Cleveland State University, Cleveland, Ohio, United States of America; University of Chicago, United States of America

## Abstract

The Hedgehog (HH) signaling pathway is critical for normal embryonic development, tissue patterning and cell differentiation. Aberrant HH signaling is involved in multiple human cancers. HH signaling involves a multi-protein cascade activating the GLI proteins that transcriptionally regulate HH target genes. We have previously reported that HH signaling is essential for human colon cancer cell survival and inhibition of this signal induces DNA damage and extensive cell death. Here we report that the HH/GLI axis regulates human telomerase reverse transcriptase (hTERT), which determines the replication potential of cancer cells. Suppression of GLI1/GLI2 functions by a C-terminus truncated GLI3 repressor mutant (GLI3R), or by GANT61, a pharmacological inhibitor of GLI1/GLI2, reduced hTERT protein expression in human colon cancer, prostate cancer and Glioblastoma multiforme (GBM) cell lines. Expression of an N-terminus deleted constitutively active mutant of GLI2 (GLI2ΔN) increased hTERT mRNA and protein expression and hTERT promoter driven luciferase activity in human colon cancer cells while GANT61 inhibited hTERT mRNA expression and hTERT promoter driven luciferase activity. Chromatin immunoprecipitation with GLI1 or GLI2 antibodies precipitated fragments of the hTERT promoter in human colon cancer cells, which was reduced upon exposure to GANT61. In contrast, expression of GLI1 or GLI2ΔN in non-malignant 293T cells failed to alter the levels of hTERT mRNA and protein, or hTERT promoter driven luciferase activity. Further, expression of GLI2ΔN increased the telomerase enzyme activity, which was reduced by GANT61 administration in human colon cancer, prostate cancer, and GBM cells. These results identify hTERT as a direct target of the HH signaling pathway, and reveal a previously unknown role of the HH/GLI axis in regulating the replication potential of cancer cells. These findings are of significance in understanding the important regulatory mechanisms that determine the functions of HH/GLI signaling in cancer cells.

## Introduction

Classical HH signaling initiates when the soluble HH ligands, Sonic (SHH), Desert (DHH) or Indian (IHH) HH bind their transmembrane receptor Patched (Ptch), thereby releasing the transmembrane protein, Smoothened (Smo) from Ptch-mediated inhibition. Smo subsequently activates the GLI family of transcription factors that regulate HH target genes. The GLI family of transcription factors includes GLI1, GLI2 and GLI3. By virtue of a C-terminal activator and N-terminal repressor domains, GLI2 and GLI3 have context-dependent activator or repressor activity. GLI1 lacks the repressor domain and functions predominantly as an activator [1], [2]. GLI2 has a C-terminal activator and N-terminal repressor domains [3]. GLI2 is reported to be the initial mediator of HH signaling events, which then induces the expression GLI1, which further increases HH target gene expression [4]. When the HH signaling pathway is active, the latent cytoplasmic GLI proteins translocate to the nucleus where they bind the GACCACCCA-like elements on the promoters of the HH-target genes [5], [6]. HH signaling regulates cellular events by modulating specific target genes. During normal embryonic development, HH signaling activity is essential, being regulated spatially and temporally resulting in normal tissue patterning and differentiation. Coordinated HH signaling is also involved in cellular proliferation and survival, maintenance of stemness and determination of cell fate [6]. Aberrantly activated HH signaling is involved in multiple human cancers and it regulates cancer cell proliferation, survival, cancer stem cell functions, epithelial to mesenchymal transition and metastasis [6]. We have reported that HH signaling is critical for the survival of human colon cancer cells, while blocking these signals induces rapid DNA damage, culminating in extensive cytotoxicity [7], [8], [9], [10]. Unlimited replication potential of cancer cells is closely associated with cancer cell survival, however, the role of HH signaling in the replication potential of cancer cells is not known.

Replication potential of human somatic cells is limited by special heterochromatic structures known as telomeres at the ends of linear chromosomes [11]. Mammalian telomeres are comprised of tandem repeats of TTAGGG sequences that are subjected to shortening with every DNA replication cycle [12]. Conventional DNA polymerases are not capable of fully replicating the ends of linear DNA molecules; hence, telomeric DNA is expected to shorten with every DNA replication cycle. Critically shortened telomeres fail to protect chromosomal ends resulting in irreversible growth arrest and limited cellular lifespan. Hence, telomere homeostasis is critical for cell proliferation and survival. Telomerase, a ribonucleoprotein comprised of a RNA component (TR) and a reverse transcriptase catalytic subunit (TERT), replenishes the telomere repeats and hence regulates cellular replicative potential [13]. In most adult cells, TR is constitutively present but TERT expression is repressed, resulting in limited proliferation potential and cellular life span [14], [15]. In actively proliferating cells such as stem cells and cancer cells, TERT expression is upregulated resulting in unlimited replicative potential and immortality of these cells [16]. Human TERT (hTERT) expression and activity has been evidenced in >75% of human colorectal cancer cells, but only 3–15% of normal mucosa and surrounding non-cancerous cells [17]. In concert with its importance in cancer cell survival, hTERT is stringently regulated with multiple activators and repressors, of which several have been identified.

Here we demonstrate for the first time that HH signaling trancriptionally upregulates hTERT. Suppression of GLI1/GLI2 reduced hTERT protein levels in human colon, prostate and brain cancer cells. Overexpression of GLI2ΔN increased the levels of hTERT mRNA, protein and hTERT promoter-driven luciferase (luc) activity in colon cancer cells. Blocking GLI1/2 activity reduced hTERT mRNA expression and the direct interaction between GLI1/GLI2 proteins and the hTERT promoter in human colon cancer cells. In contrast, GLI1/GLI2ΔN expression in non-cancerous 293T cells did not alter the levels of hTERT mRNA, protein or hTERT promoter-luc activity. Abrogating HH signaling in cancer cells decreased the telomerase activity, which was increased by GLI2ΔN expression. These results demonstrate hTERT to be a transcriptional target of the HH signaling pathway and identify a previously unknown role of the HH/GLI axis in regulating the replication potential of cancer cells. These findings reveal a new function of HH signaling in increasing the replicative ability of cancer cells. Of interest, HH signaling regulates hTERT in a context-dependent manner in human cancer cells, in contrast to non-malignant cells, which may have implications in cancer therapeutics.

## Materials and Methods

### Cell Culture and Reagents

HT29, SW480, HCT116 and 293T cells were obtained from American Type Culture Collection. C4-2, DU145, and PC3 cells were kind gifts of Dr. Alexandru Almasan (Cleveland Clinic, OH). U87 cells were a kind gift of Dr. Candece Gladson (Cleveland Clinic, OH). The cells were routinely verified by microscopic analysis of cell morphology, growth characteristics, and response to cytotoxic agents [Annexin V/propidium iodide (PI) staining]. cDNA microarray gene profiles were also characteristic and cells were verified biannually to be mycoplasma-free. HT29, HCT116, SW480, C4-2, DU145 and PC3 cells were maintained in 10% FBS-supplemented RPMI medium while U87 and 293T cells were maintained in 10% FBS-supplemented DMEM. The cells were trypsinized and counted using a Z2 Coulter particle count and size analyzer (Beckman Coulter). For Western analysis, antibody against HSP90α/β was purchased from Santa Cruz Biotechnology; anti-GLI1 and anti-hTERT antibodies were from Novus Biologicals, and anti-GLI2 antibody was from Cell Signaling Technology. Anti-c-myc antibody (9E10) was obtained from the Hybridoma Core, Lerner Research Institute. GANT61 was purchased from Calbiochem. Dr. Graham W. Neill (Queen Mary University of London, UK) kindly provided the GLI1 and GLI2ΔN plasmids in pBabe-Puro (pBP) mammalian expression vector. Full-length hTERT-pBP plasmid was a gift of Dr. Robert Weinberg (Addgene plasmid #1771).

### Western Blot Analysis

Total cellular lysates were prepared using RIPA lysis buffer (Cell Signaling Technology). Protein (60 µg) was resolved on 10% or 5% SDS-PAGE gels. Separated proteins were transferred to polyvinylidene difluoride membranes and subsequently blocked in blocking buffer [5% nonfat dry milk in 1X Tris Buffer Saline with 0.1% Tween 20 (TBS-T)] for 1 hour. Membranes were washed in 1X TBS-T, incubated with primary antibody overnight at 4°C, washed and incubated with secondary antibody for 1 hour, and finally developed using Super Signal Pico substrate from Pierce Biotechnology.

### RNA Isolation and mRNA Analysis

Total RNA was isolated using the Qiagen RNeasy mini kit according to the manufacturer’s protocol. Total RNA was converted to cDNA using random primers (iScript Select cDNA synthesis kit; BIO-RAD), and used for real-time mRNA expression analysis using 40 cycles of Applied Biosystems 7500 Real-Time PCR instrumentation and software. Primers were designed using NCBI/Primer-BLAST and used to generate the PCR products. The GLI1, GLI2 and GAPDH primers were previously described [9].

hTERT forward primer: 5′-CCTGGGTGGCACGGCTTTTGTTC-3′.

hTERT reverse primer: 5′-CAGCCTTGAAGCCGCGGTTGA-3′.

### GLI3R and Transient Transfections

The myc-tagged C-terminus deleted construct GLI3R (gift of Dr. Ariel Ruiz i Altaba, University of Geneva Medical School, Geneva, Switzerland) has been previously described (3). HT29 cells were transiently transfected using Lipofectamine 2000.

(Invitrogen) with GLI3R or the empty vector pCS2-MT (gifted by Dr. David Turner, at The Molecular & Behavioral Neuroscience Institute, University of Michigan, Ann Arbor, MI). Cells were used for experiments 24 hr, 48 hr, or 72 hours posttransfection.

### Chromatin Immunoprecipitation (ChIP) Analysis

The cells treated with GANT61 (20 µM) for 24 hr were cross-linked in 1% formaldehyde/PBS, 10 min, 37°C, which was terminated in glycine, 5 min. Cells were washed in PBS and nuclear extracts prepared. Nuclei were sonicated to generate chromatin fragments (≈ 500 to 800 bp). The chromatin was precleared with a mixture of proteinA-sepharose and proteinG-sepharose that was blocked with bovine serum albumin (1 mg/ml) and Salmon sperm DNA (1 mg/ml). 10% of the precleared chromatin was used as input control. Equal quantities of the precleared chromatin fragments were immunoprecipitated with antibodies specific for GLI1 (Novus Biologicals, CO), GLI2 (Cell Signaling Technology (MA), IgG (Abcam, MA; negative control), or histone H3 (Abcam, MA; positive control). Methods were performed as described previously (3, 4). The immunoprecipitated products were washed extensively and eluted. 30 µl of the eluted products were treated with RNase A and Proteinase K followed by reverse cross-linking and DNA isolation. Gene specific primers quantified the amounts of hTERT and BCL-2 promoter DNA in the immunoprecipitated fractions. PCR products were resolved on a 1% agarose gel, stained with ethidium bromide, and visualized under UV light.

The primers used for ChIP analysis are as follows:

hTERT promoter forward: 5′-TGATGGGGACCGTTCCTTCCATC-3′.

hTERT promoter reverse: 5′-ACACGGCCCACCCAGGGTTTA-3′.

BCL2-promoter forward: 5′-CCGGACGCGC CCTCCC-3′.

BCL-2 promoter reverse: 5′-GGTGCCTGTCCTCTTACTTCATTCTC-3′.

### Luciferase Assay

The full-length hTERT promoter (−3337/+438), and upstream deletion mutants (−1226/+438 and −233/+438) -driven luciferase reporter constructs were kindly provided by Dr. Ralf Janknecht, University of Oklahoma Health Sciences Center, OK [18]. HT29 or 293T cells were transiently transfected using Lipofectamine 2000 (Invitrogen) with 4 mg of the luciferase reporter and 0.4 mg pRLTK (renilla luciferase driven by TK promoter). 24 hr after transfection, cells were analyzed using the dual luciferase kit (Promega Corporation) according to the manufacturer’s protocol. Luciferase activity was detected using Victor2 multilabel counter, and normalized to renilla luciferase activity as a control for transfection efficiency.

### Telomerase Repeat Amplification Protocol (TRAP) Assay

Cells were lysed in 1X CHAPS lysis buffer and 0.25 ug of the lysates were used to perform TRAP assay. 1 µg of TS primer was end-labeled with 3 µl of γ ^P32^ATP (Perkin Elmer) using 1 µl of PNK (NEB) in a 20 µl reaction at 37°C for 45 min and purified through a Sephadex G-25 column. In each reaction, 2 pmole of γ-32P end labeled TS primers, and reverse primers for PCR amplification were mixed with 0.05 mM of dNTP, 20 mM Tris•Cl pH 8.3, 1.5 mM MgCl_2_, 63 mM KCl, 0.05% Tween 20, and 1 mM EGTA. 20 ng of RNase A was added with the cell lysate in reactions treated with RNase. Telomerase-mediated primer extension was carried out at 30°C for 30 min followed by PCR amplification using the TS and reverse primers. Products from TRAP assays were analyzed on 12.5% non-denaturing PAGE. The TIFF files of the scanned gel images were quantified by densitometry using the Image J software and the telomerase activity was determined using the formula mentioned in the manufacturer’s protocol for the TRAPEZE Telomerase detection kit (Chemicon International Inc., MA).

### Statistical Analysis

All the statistical analyses were performed using Prism (GraphPad Software, La Jolla, CA).

## Results

### HH Signaling Upregulates hTERT Expression in Human Cancer Cells

**Figure 1:**
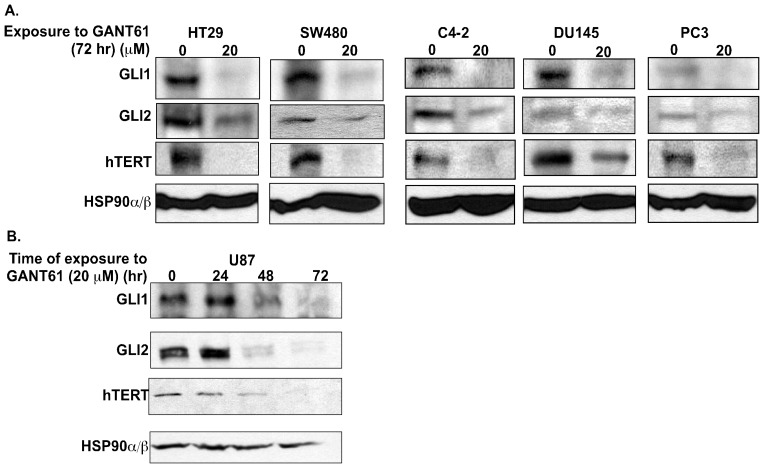
Pharmacologic inhibition of GLI1/GLI2 results in decreased hTERT protein expression in human cancer cells. ***A:*** HT29, SW480 (colon carcinoma cells), C4-2, DU145 and PC3 (prostate cancer cells) were treated for 72 hr with either DMSO (vehicle control) or GANT61 (20 µM). ***B:*** U87 (GBM cells) were treated with GANT61 (20 µM) for 0–72 hr. Steady state levels of GLI1, GLI2 and hTERT protein were determined by Western analysis. HSP90α/β was used as loading control.

**Figure 2:**
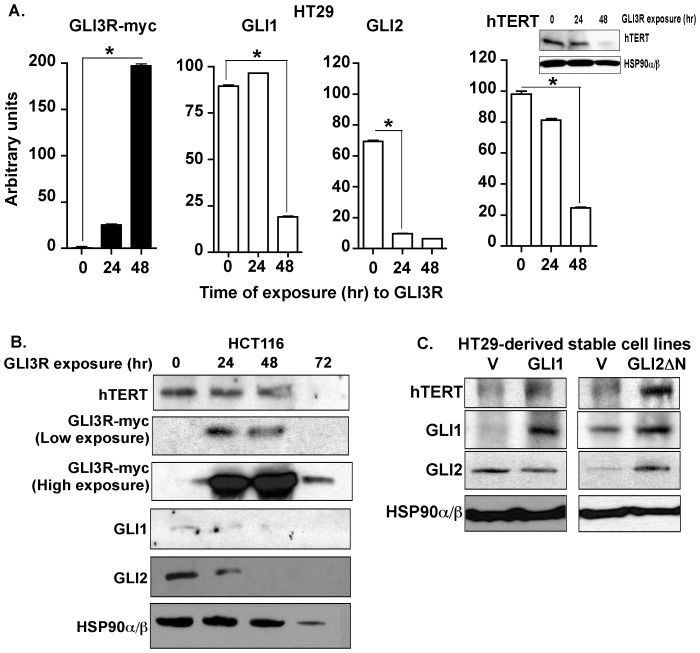
Genetic regulation of HH signaling modulates hTERT expression in human colon cancer cells. ***A:*** HT29, ***B:*** HCT116 cells were transiently transfected with GLI3R (myc-tagged) for 48 hr or 72 hr, respectively. Steady state levels of GLI1, GLI2 and hTERT were determined by Western analysis represented as densitometry of the blots in ***A*** with one representative hTERT blot (inset). Expression of GLI3R was determined using anti-myc antibody. ***C:*** pBP empty vector (V) or full length GLI1 cDNA in pBP (GLI1) or GLI2ΔN in pBP (GLI2ΔN) was stably expressed into HT29 cells, and cells were cultured for 10 passages. Steady state levels of GLI1, GLI2 and hTERT were measured by Western blot. HSP90α/β was used as a loading control. *p<0.0001.

Dysregulated HH signaling is known to promote cell proliferation, cell cycle progression and cell survival in human cancer cells, hence it was considered that the activated HH pathway may also play a role in the replication potential of cancer cells. Telomerase, a key regulator of the replication potential and closely linked with cellular proliferation and survival, is therefore a strong candidate for regulation by activated HH signaling in cancer cells. To test this hypothesis, the relationship between the HH/GLI signaling axis and telomerase expression was examined in multiple human cancer cell lines. We abrogated the HH/GLI signaling axis in multiple human cancer cell lines with pharmacological inhibitors and measured telomerase expression. Human colon cancer cells HT29 and SW480, exposed to GANT61 (20 µM), a small molecule inhibitor of GLI1 and GLI2 [9], for 72 hr demonstrated reduced steady state levels of GLI1, GLI2 and hTERT proteins (Fig. 1A). Similarly, blocking HH/GLI signaling in the prostate cancer cells C4-2, DU145 and PC3 also demonstrated decreased GLI1, GLI2 and hTERT protein expression (Fig. 1A). Administration of GANT61 (20 µM) to the GBM cell line U87 over a period of 72 hr resulted in reduced GLI1, GLI2 and hTERT protein expressions (Fig. 1B). We further validated the effects of HH signaling pathway on hTERT expression by genetically modifying the HH/GLI signaling pathway. We employed a C-terminus deleted mutant of GLI3 (GLI3R, myc-tagged GLI3C’DCla1 [3]), which represses GLI1 and GLI2 activity [8]. Following transient transfection of GLI3R into HT29 (densitometric quantification in Fig. 2A) and HCT116 (Fig. 2B) colon cancer cell lines, GLI1, GLI2 and hTERT protein levels were decreased over a period of 48 hr – 72 hr. Conversely, stable expression of either GLI1 or GLI2ΔN, an N-terminus deleted mutant of GLI2, with constitutive activator function in HT29 cells for 10 passages demonstrated increased hTERT protein expression (Fig. 2C).

### GLI Transcription Factors Interact with the hTERT Promoter in Human Cancer Cells

**Figure 3:**
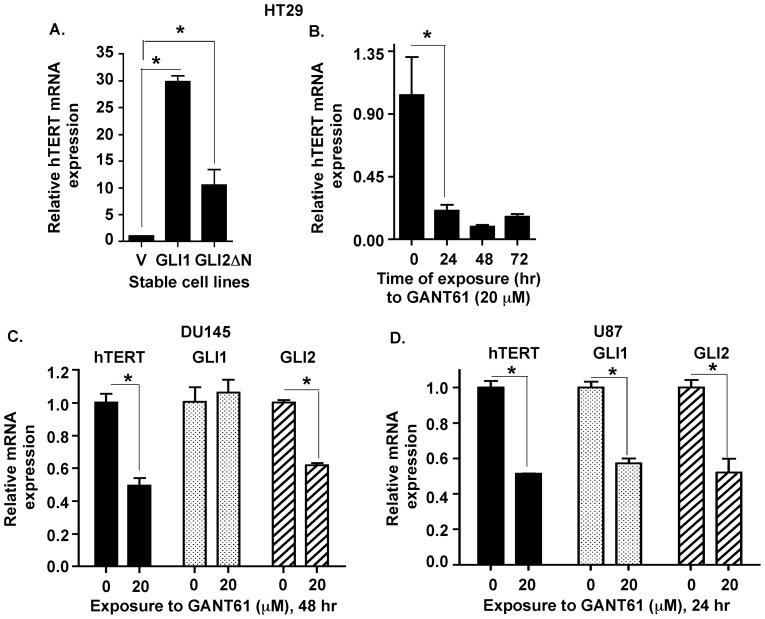
HH/GLI signaling regulates hTERT mRNA expression. ***A:*** HT29 cells stably expressing empty vector (V), GLI1 (GLI1) or GLI2ΔN (GLI2ΔN) were analyzed for hTERT, GLI1 and GLI2 mRNA expression determined by Real-Time PCR. ***B:*** Exposure of HT29 cells to GANT61 (20 µM; 0–72 hr) reduced expression of hTERT mRNA, determined by Real-Time PCR. ***C:*** DU145, ***D:*** U87 cells were exposed to GANT61 (20 µM; 48 hr or 24 hr respectively) and GLI1, GLI2 and hTERT mRNA was measured by Real-time PCR. Data represent the mean ± SD of 3 determinations. *p<0.05.

Next, we investigated the mechanism by which HH signals regulate the expression of hTERT in human cancer cells. Transcriptional regulation of hTERT expression is a common mechanism of hTERT upregulation in cancer cells [19]. Since the GLI proteins are transcription factors, we hypothesized that GLI1 and GLI2 transcriptionally regulate hTERT expression in cancer cells. hTERT mRNA expression was elevated in both GLI1- and GLI2-overexpressing HT29 cells (Fig. 3A). When HT29 cells were exposed to GANT61 (20 µM), hTERT mRNA levels were significantly decreased within 24 hr and remained suppressed through 72 hr (Fig. 3B). DU145 cells demonstrated decreased GLI2 and hTERT mRNA levels when exposed to GANT61 (20 µM) while GLI1 transcript expression remained unaltered at 48 hr post-treatment (Fig. 3C). Upon exposure to GANT61 (20 µM), U87 cells demonstrated reductions in GLI1, GLI2 and hTERT mRNA within 24 hr (Fig. 3D). These results confirmed transcriptional regulation of hTERT expression by HH signaling pathway in human cancer cells.

**Figure 4:**
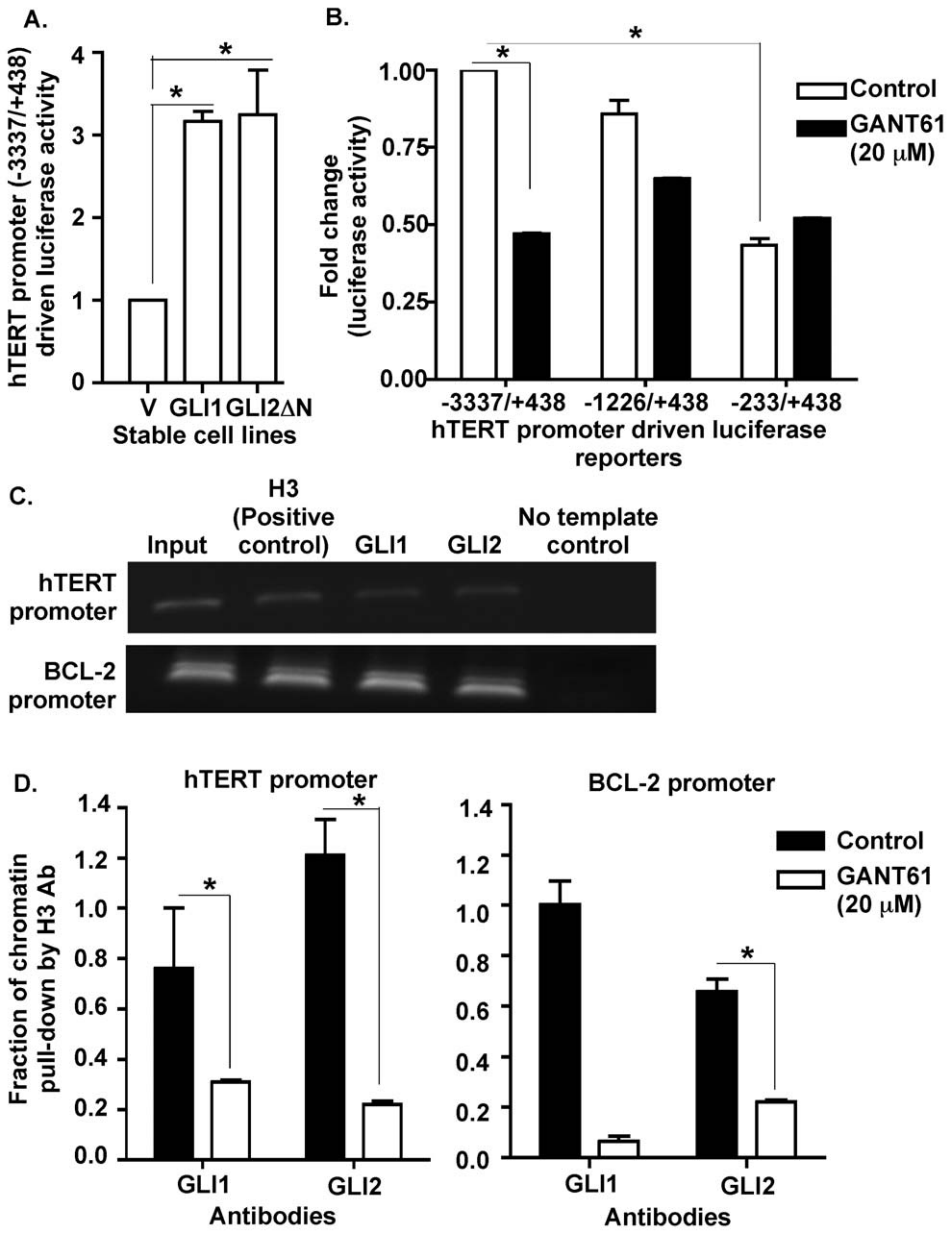
GLI1 and GLI2 proteins regulate hTERT promoter activity. ***A:*** Stable derivatives of HT29 cells (described in Fig. 2C) were co-transfected with a full-length hTERT promoter driven luciferase (−3337/+438) reporter and renilla luciferase (pRL-TK) constructs for 24 hr. Lysates were prepared, and luciferase activity determined as described in Materials and Methods. hTERT promoter luciferase activity was normalized against renilla luciferase activity and is presented as mean ± SD, n = 3. ***B:*** HT29 cells were co-transfected with either the full length (−3337/+438) or upstream deleted mutants (−1226/+438 or −233/+438) of hTERT prom-luc reporters and pRL-TK followed by exposure to GANT61 (20 µM, 24 hr) and determination of luciferase activity. hTERT promoter luciferase activity was normalized against renilla luciferase activity and is presented as mean ± SD, n = 3. ***C:*** HT29 cells were employed for ChIP analysis using antibodies specific for GLI1, GLI2, or histone H3 (positive control, used for normalization). ***D:*** HT29 cells treated with GANT61 (20 µM, 24 hr) were similarly evaluated by ChIP analysis. Subsequent Real-Time PCR used primers that flanked the promoter regions of hTERT or the GLI target gene, BCL-2 (positive control). *p<0.05.

To dissect the mechanism of transcriptional regulation of hTERT expression by HH signaling pathway, we monitored the effects of HH signaling on hTERT promoter activity in HT29 cells. The full-length hTERT promoter-driven luciferase (FL hTERT prom-luc) reporter and pRL-TK was transiently co-transfected into HT29-derived stable cell lines over-expressing GLI1 or GLI2 or hTERT. Both GLI1 and GLI2ΔN expressing cells demonstrated a 4-fold increase in hTERT promoter activity within 24 hr of transfection (Fig. 4A). To identify the minimal promoter length required for the HH-dependent effects on hTERT promoter activity, the FL hTERT promoter (−3337/+438), and upstream deletion mutants (−1226/+438 and −233/+438) -driven luciferase reporters were co-transfected into HT29 cells followed by exposure to either vehicle control or GANT61 (20 µM) for 24 hr. Data demonstrate that the −1226/+438 region is the minimal requirement for HH-dependent hTERT promoter activation although, the effects are less than that of the FL hTERT promoter (Fig. 4B). The FL hTERT promoter activity was significantly reduced upon blocking GLI activity with GANT61 (20 µM) (Fig. 4B). Next, we investigated whether the GLI transcription factors directly interacted with the hTERT promoter in cancer cells. *In silico* analysis of the hTERT promoter revealed 7 putative binding sites for the GLI family of transcription factors. Both GLI1 and GLI2 antibodies precipitated fragments of the hTERT promoter in ChIP analyses (Fig. 4C). PCR amplification of the chromatin fragments using primers specific for hTERT promoter regions resulted in amplicons that contained atleast the core (−226/+360) hTERT promoter verified by sequencing the PCR amplicons. Binding of GLI1 and GLI2 to the hTERT promoter was reduced in the presence of GANT61 (20 µM) within 24 hr (Fig. 4D). BCL-2, previously reported as a target of both GLI1 and GLI2, was used as positive control (Fig. 4C and 4D).

### HH/GLI Signaling does not Regulate hTERT Expression in Non-malignant 293T Cells

**Figure 5:**
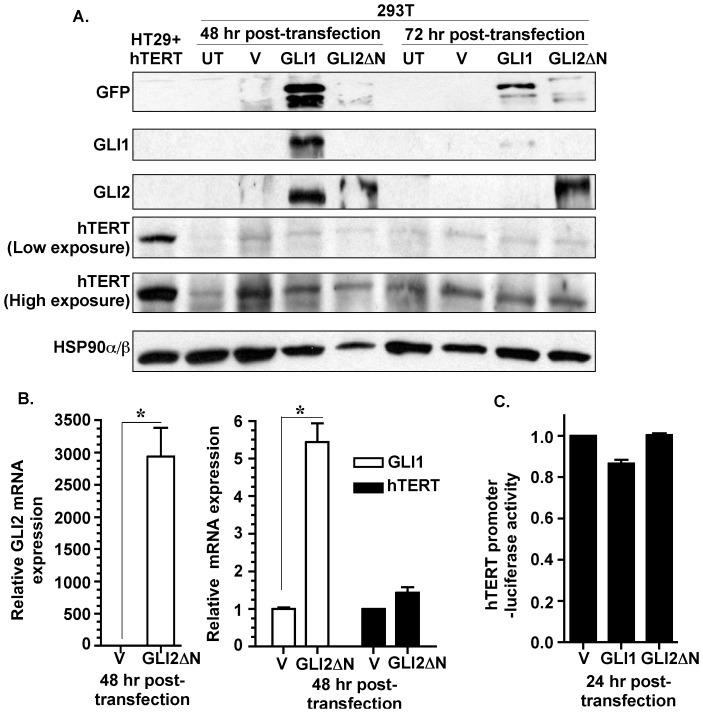
Over-expression of GLI1 or GLI2 in non-malignant 293T cells does not influence hTERT expression. ***A:*** 293T cells were untreated (UT), transiently transfected with empty vector (V) or GFP-tagged GLI1 (GLI1) or GFP-tagged GLI2ΔN (GLI2ΔN). Expression of GLI1, GLI2 and hTERT was determined for 48 and 72 hrs by Western analysis. HSP90α/β was used as loading control and GFP was used to mark exogenously expressed GFP-tagged GLI proteins. Total cell lysate from HT29 cells stably expressing hTERT (HT29+hTERT) served as positive control. ***B:*** 293T cells transiently transfected with vector or GLI2 (as described in Figure 5A) were analyzed for GLI1, GLI2 and hTERT mRNA expression via qPCR. Data represent the mean ± SD of 3 determinations. ***C:*** 293T cells transiently co-transfected with FL-hTERT prom-luc, renilla luceferase reporters and vector, GLI1 or GLI2 (as described in Figure 5A). 24 hr post-transfection the cells were analyzed for luciferase activity. hTERT promoter-driven luciferase activity was normalized against renilla luciferase activity and is represented as mean ± SD, n = 3. *p<0.05.

We further investigated whether HH signaling transcriptionally regulates hTERT in non-malignant cells. 293T cells are experimentally transformed but lack the ability to readily form tumors in nude mice [20] and demonstrate an inducible HH/GLI signaling axis [21]. We transiently transfected 293T cells with, either GLI1 or GLI2ΔN expression plasmids, and measured hTERT expression by western analysis. Although GLI1 and GLI2 protein expressions were significantly elevated in the transfected 293T cells, hTERT protein expression remained unaltered at 48 hr and 72 hr (Fig. 5A). hTERT over-expressing HT29 cells were used as a positive control for hTERT protein analysis (Fig. 5A). We measured hTERT transcript following transient transfection of GLI2ΔN into 293T cells. Within 48 hr of transfection, 293T cells demonstrated robust increase in GLI2 mRNA and >5 fold increase in GLI1 mRNA levels, but no significant elevation in hTERT mRNA level (Fig. 5B). Further, transient co-transfection of the FL hTERT prom-luc reporter and either GLI1 or GLI2ΔN expression plasmids did not increase the luciferase activity in 293T cells (Fig. 5C). These findings underscore the context-dependent functions of HH signaling pathway that selectively upregulates hTERT expression in malignant cells in contrast to non-malignant cells.

### HH/GLI Signaling Regulates hTERT Enzymatic Activity in Human Cancer Cells

**Figure 6:**
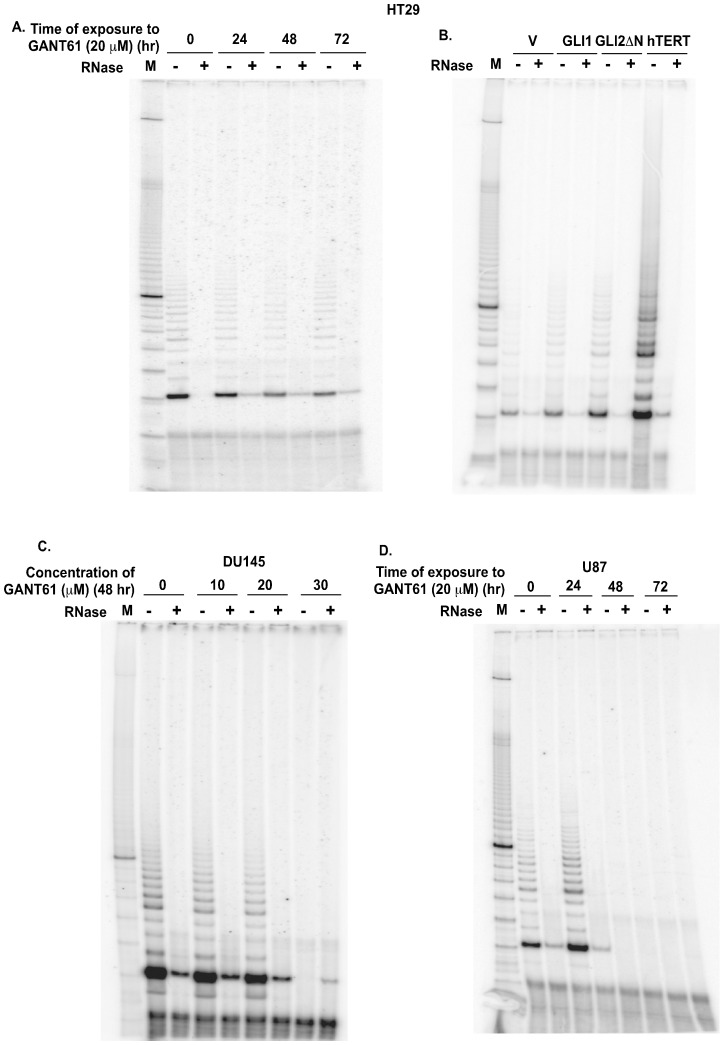
The HH/GLI signaling pathway regulates telomerase activity. ***A:*** HT29 cells were treated with GANT61 (20 µM) for 0–72 hr, and lysates were extracted at intervals for TRAP analysis. 100 bp DNA ladder was used as a molecular marker (M). ***B:*** HT29 cells stably expressing vector (V), GLI1 or GLI2 (GLI2ΔN) (described in Figure 3A) were evaluated for telomerase activity by TRAP assay. hTERT over-expressing cells were used as positive control. ***C:*** U87 and ***D:*** DU145 cells were treated with GANT61 (0–30 µM; 0–72 hr) and telomerase activity was analyzed by the TRAP assay.

Aberrant upregulation of hTERT expression is associated with increased telomerase reverse transcriptase enzyme activity in cancer cells. Hence, we tested the functional significance of the HH/GLI/hTERT axis by measuring telomerase enzyme activity in cancer cells. TRAP assay was used to determine the activity of hTERT upon altering the HH/GLI signaling cascade. GANT61 (20 µM) administration led to reduced telomerase activity by 48 hr, which was sustained for up to 72 hr in HT29 cells (Fig. 6A, quantified in Fig. S1). Conversely, stable derivatives of HT29 cells expressing GLI2ΔN demonstrated increased telomerase activity in comparison to the vector control, while GLI1 over-expressing cells had a modest increase in telomerase activity (Fig. 6B, quantified in Fig. S1). hTERT over-expressing cells with elevated telomerase activity served as a positive control (Fig. 6B). Telomerase activity was reduced in DU145 cells exposed to GANT61 (0–30 µM) for 48 hr (Fig. 6C). U87 cells also demonstrated reductions in telomerase activity within 48 hr of exposure to GANT61 (20 µM), which was sustained till 72 hr (Fig. 6D). These observations demonstrate that HH signaling upregulates both the hTERT expression and telomerase activity in human cancer cells.

## Discussion

HH signaling activity is essential for normal embryonic development where it regulates cell differentiation and organ formation in a gradient-dependent manner [22]. HH signaling regulates cellular proliferation by transcriptionally modulating genes that control cell cycle progression such as cyclin D, cyclin E and, also by Ptch-mediated sequestration of cyclin B in the cyctoplasm [23], [24], [25]. In the adult stage, active HH signaling persists in a small subset of cells that confer regenerative potential for the mature organs [26]. Dysregulation of HH signaling has been reported in multiple human cancers, including basal cell carcinoma [27], [28], medulloblastoma [1], [29], rhabdomyosarcoma [30], glioma [1], pancreatic adenocarcinoma [31], [32], [33], prostate cancer [34] and colon carcinoma [7], [8], [9], [10]. In cancer cells, autocrine ligand-dependent and oncogene-driven ligand-independent mechanisms maintain an active HH signaling cascade. Aberrant HH activity drives tumor formation and tumor maintenance by inducing pro-survival signals and blocking apoptotic signals in cancer cells [9], [35]. Another hallmark of cancer is unlimited proliferation potential of cancer cells resulting in immortality, however a link to dysregulated HH signaling was not known.

Telomeres are specialized nucleoprotein structures at the chromosomal ends that are important for chromosomal stability and immortality of cells. Normal somatic cells encounter a gradual attrition of telomere lengths with every DNA replication cycle thereby limiting cellular lifespan. Normal stem cells and cancer cells escape this check and replenish their telomere lengths by increased telomerase activity. Telomerase is a ribonucleoprotein comprised of human telomerase reverse transcriptase enzyme, hTERT and telomerase RNA, hTR [36], [37]. In cancer cells, hTERT expression is aberrantly restored leading to increased telomerase activity that maintains telomere length and confers unlimited replication potential. hTERT expression and telomerase activity has a close association [33] with human cancers [17], [38]. Transformation of telomerase-negative normal cells in vitro requires hTERT expression [39], delineating hTERT expression as a rate-limiting step in telomere length homeostasis and cellular transformation. The regulation of hTERT expression has been extensively studied and numerous positive and negative regulators have been identified [40], [41].

In this study, we have shown for the first time that the HH signaling pathway ensures limitless replication potential of cancer cells by regulating hTERT expression and activity. In multiple human cancer cell lines including colon, prostate and brain, suppression of GLI1 and GLI2 expression using GANT61, a small molecule inhibitor resulted in reduced expression of hTERT mRNA, protein and enzyme activity. This finding indicated a regulatory axis between HH signaling and hTERT in human cancer cells. Using a genetic approach to suppress GLI1 and GLI2 using GLI3R, hTERT expression could also be inhibited in colon cancer cells. Forced expression of GLI1 or a constitutively active mutant of GLI2 expression increased hTERT mRNA and protein in human colon cancer cells demonstrating HH/GLI-mediated regulation of hTERT expression. hTERT promoter-driven luciferase reporter activity was significantly reduced upon exposure to GANT61. Further, hTERT promoter activity was significantly elevated in human colon cancer cells stably over-expressing GLI1 or GLI2. *In silico* analysis of the hTERT promoter revealed 7 putative binding sites for the GLI family of transcription factors suggesting a direct transcriptional mode of regulation of hTERT by GLI. ChIP analysis demonstrated that GLI1 and GLI2 proteins physically interacted with the hTERT promoter and this interaction was sensitive to GANT61-mediated inhibition of GLI1 and GLI2 activity. We have previously reported that the GLI transcription factors are activated in a SMO-independent manner via oncogenic pathways such as RAS/RAF signaling axis in human colon cancer cells that are hence more sensitive to inhibition of GLI instead of SMO [7], [8]. Hence, for the studies described here, the HH signaling axis was blocked at the level of the GLI transcription factors using GANT61 (a small molecule inhibitor of GLI1/2 activity) instead of SMO inhibitors. Collectively, these findings demonstrated an HH/GLI/hTERT axis in human cancer cells.

Recent reports identified a direct link between the Wnt/β-catenin signaling pathway and hTERT expression in normal embryonic and adult stem cells and cancer cells [42], [43], [44]. Interestingly, our data demonstrate that there is a context-dependent regulation of the hTERT promoter by the HH/GLI signaling pathway. The HH/GLI signaling axis increased hTERT expression in the panel of colon, prostate and brain cancer cells investigated here, in contrast to non-malignant 293T cells, where hTERT expression was unresponsive to HH/GLI activity. This underscores the differential regulation of genes in normal versus noeplastic cells. Recent evidences suggest that the hTERT promoter is regulated by epigenetic modification. For example, SYMD3, a histone methyltransferase upregulated and implicated in oncogenesis, directly binds the hTERT promoter and serves as a licensing factor for hTERT transcription by enabling the interaction of other transcription factors [45]. Such epigenetic regulators may determine context-dependent regulation of hTERT expression and activity by various transcription factors. It remains to be determined whether HH/GLI signaling activity at the hTERT promoter is dependent on such epigenetic regulators. However, context-dependent functional heterogeneity is a well-known characteristic of the HH signaling pathway [3]. By virtue of harboring activator and repressor functions, the GLI proteins can elicit a context-dependent positive or negative impact on cellular functions. GLI1 lacks the amino-terminal repressor domain and serves as an activator [46]. GLI2, which has an amino-terminal repressor domain and a carboxy-terminal activator domain, functions as a repressor in its full-length form and upon truncation of its amino-terminus converts to a transcriptional activator in cells. GLI3 also possesses amino- and carboxy- termini but functions predominantly as a transcriptional repressor [47]. The three GLI proteins co-operate in HH-responsive cells to integrate the HH signals in conjunction with other cellular signals. The net result of the collective activator and repressor functions of GLI1, GLI2 and GLI3 dictate the status of the GLI transcription program which modulate specific HH target genes in cells [48]. HH/GLI signaling can function in a species-dependent manner, eg. GLI1 mediates SHH-induced differentiation of frog neural plate, which is blocked by GLI2 and GLI3 activity [49]. Positive or negative activity of the GLI proteins is developmental stage- and target-dependent [49]. In mice, GLI2 and GLI3 transcription factors have essential and partially overlapping functions while GLI1 is dispensable [50]. Such observations emphasize the context-dependent roles of the HH/GLI axis in regulating target genes. Binding partners such as Zic proteins also regulate the functions of the GLI transcription factors in a context-dependent manner [51]. GLI transcriptional activity is also regulated by the acetylation status of GLI proteins [52].

In summary, our results demonstrate that HH signaling regulates hTERT, which determines the replication potential of cancer cells. Suppression of both GLI1 and GLI2 functions reduced hTERT mRNA and protein expression in human colon, prostate and brain cancer cell lines. Exogenous expression of GLI1 or GLI2 increased hTERT mRNA and protein expressions and hTERT promoter-luciferase activity in human colon cancer cells. Blocking GLI transcriptional function reduced the GLI protein-hTERT promoter interaction. Exogenous expression of GLI1 or GLI2ΔN in the non-malignant 293T cells failed to alter the levels of hTERT mRNA and protein, or hTERT promoter-luciferase activity. Blocking GLI transcriptional activity reduced the telomerase activity in human colon, prostate and GBM cells, while stable over-expression of GLI2 in HT29 cells increased telomerase activity. Collectively, our data reveal a previously unknown function of the HH/GLI axis in controlling unlimited replication potential of cancer cells by transcriptionally regulating hTERT gene expression, a critical determinant of the life span of cancer cells.

## Supporting Information

Figure S1 **Densitometric analysis of telomerase activity in HT29 cells.** Telomerase enzyme activity was quantified densitometrically in HT29 cells treated with GANT61 (20 µM) for 0–72 hr ***(A)*** and HT29 cells stably expressing vector (V), GLI1 or GLI2 (GLI2ΔN) ***(B)*** as described in Materials and Methods. Data is represented as mean ± SD, n = 3. *p<0.05.
(TIF)
